# Overlapping and non-overlapping roles of the class-I histone deacetylase-1 corepressors LET-418, SIN-3, and SPR-1 in *Caenorhabditis elegans* embryonic development

**DOI:** 10.1007/s13258-021-01076-1

**Published:** 2021-03-19

**Authors:** Yukihiro Kubota, Yuto Ohnishi, Tasuku Hamasaki, Gen Yasui, Natsumi Ota, Hiromu Kitagawa, Arashi Esaki, Muhamad Fahmi, Masahiro Ito

**Affiliations:** 1 Department of Bioinformatics, College of Life Sciences, Ritsumeikan University, 1-1-1 Nojihigashi, Kusatsu, Shiga 525-8577 Japan; 2 Advanced Life Sciences Program, Graduate School of Life Sciences, Ritsumeikan University, 1-1-1 Nojihigashi, Kusatsu, Shiga 525-8577 Japan

**Keywords:** *C. elegans*, Corepressor, Embryo, HDAC, RNA-seq, Comparative transcriptome

## Abstract

**Background:**

Histone deacetylase (HDAC)-1, a Class-I HDAC family member, forms three types of complexes, the nucleosome remodeling deacetylase, Sin3, and CoREST complexes with the specific corepressor components chromodomain-helicase-DNA-binding protein 3 (Mi2/CHD-3), Sin3, and REST corepressor 1 (RCOR1), respectively, in humans.

**Objective:**

To elucidate the functional relationships among the three transcriptional corepressors during embryogenesis.

**Methods:**

The activities of HDA-1, LET-418, SIN-3, and SPR-1, the homologs of HDAC-1, Mi2, Sin3, and RCOR1 in *Caenorhabditis elegans* during embryogenesis were investigated through measurement of relative mRNA expression levels and embryonic lethality given either gene knockdown or deletion. Additionally, the terminal phenotypes of each knockdown and mutant embryo were observed using a differential-interference contrast microscope. Finally, the functional relationships among the three corepressors were examined through genetic interactions and transcriptome analyses.

**Results:**

Here, we report that each of the corepressors LET-418, SIN-3, and SPR-1 are expressed and have essential roles in *C. elegans* embryonic development. Our terminal phenotype observations of single mutants further implied that LET-418, SIN-3, and SPR-1 play similar roles in promoting advancement to the middle and late embryonic stages. Combined analysis of genetic interactions and gene ontology of these corepressors indicate a prominent overlapping role among SIN-3, SPR-1, and LET-418 and between SIN-3 and SPR-1.

**Conclusion:**

Our findings suggest that the class-I HDAC-1 corepressors LET-418, SIN-3, and SPR-1 may cooperatively regulate the expression levels of some genes during *C. elegans* embryogenesis or may have some similar roles but functioning independently within a specific cell.

**Supplementary Information:**

The online version contains supplementary material available at 10.1007/s13258-021-01076-1.

## Introduction

During embryonic development, daughter cells generated from fertilized eggs contain the same genomic information as the progenitor cells when the cell division process is completed. Although they have identical genome sequences, daughter cells can differentiate from precursor cells within developing tissues and organs through the epigenomic control of gene expression. Therefore, epigenomic modifications play important roles in normal embryonic development (Cavalli 2006). Epigenomic modifications are modulated via chemical changes to histones and DNA. Acetylation and the methylation of histones affect the regulation of gene expression by influencing histone–DNA and histone–protein interactions. Histone modifications are regulated by transferases and hydrolases (Cavalli 2006). Histone acetyltransferases promote this process to neutralize the positive charge of the histone tail, thus acting as positive transcriptional regulators by weakening the physical interaction between the histone tail and DNA (Garcia-Ramirez et al. 1995). In contrast, histone deacetylases (HDACs) remove acetyl groups from histones and negatively regulate transcription by enhancing the physical interaction between the histone tail and DNA (Cosgrove et al. 2004).

The 18 human HDAC proteins are divided into four classes based on their sequence homologies with the four yeast HDAC proteins (Vaquero et al. 2007; Yang and Seto 2008). Among four class-I HDACs members, HDAC-1 forms complexes with multiple components, such as transcriptional corepressors and DNA binding proteins, and promote histone deacetylation to suppress the transcription of target genes (Hayakawa and Nakayama 2011). The HDAC-1 transcriptional corepressor forms three types of complexes in humans, the NuRD complex, Sin3 complex, and CoREST complex, with specific corepressor components (Mi2/CHD3, Sin3, and RCOR1, respectively) (Hayakawa and Nakayama 2011). These complexes are thought to function as transcriptional repressors by inhibiting the transcription of their target genes (Hayakawa and Nakayama 2011). HDACs have been implicated in regulating various vital processes, such as DNA repair, lipid metabolism, cell cycle progression, and the circadian rhythm (Feng et al. 2011; Knutson et al. 2008; Miller et al. 2010; Sun et al. 2012; Wilting et al. 2010). Furthermore, HDAC-1 proteins have been shown to play important roles in the embryogenesis of multiple model organisms (Mannervik and Levine 1999; Montgomery et al. 2007; Shi and Mello 1998; Vecera et al. 2018). However, the mechanism by which the interplay among the three HDAC-1 complexes regulates embryonic development remains unknown.

The nematode *Caenorhabditis elegans* is a model multicellular organism, for which the whole genome sequence and entire cell lineage have been completely identified (Genome sequence of the nematode *C. elegans*: a platform for investigating biology 1998; Sulston et al. 1983). Therefore, *C. elegans* is a reliable model organism to analyze the regulatory mechanism of embryogenesis. The constituents of the HDAC complex are also conserved in *C. elegans* (Wenzel et al. 2011). Human HDAC-1 shares conserved sequences with *C. elegans* ortholog of HDAC-1, HDA-1. Furthermore, *C. elegans hda-1* can help regulate vulval development (Ranawade et al. 2013). LET-418, SIN-3, and SPR-1 are *C. elegans* homologs of the human transcriptional corepressor components Mi-2/CHD3, SIN3, and RCOR1, respectively, and each corepressor has been shown to be involved in both embryonic and post-embryonic development, driving specific functions, such as male sensory cell formation, gonadal morphogenesis, and vulval development (Bender et al. 2007; Beurton et al. 2019; Choy et al. 2007; Käser-Pébernard et al. 2016; Passannante et al. 2010; Saudenova and Wicky 2018; Solari and Ahringer 2000; von Zelewsky et al. 2000). However, the functional relationships among these three transcriptional corepressors in *C. elegans* embryogenesis remain unexplored.

In this study, we identified functional similarities and differences among the transcriptional corepressors LET-418, SIN-3, and SPR-1 to understand the functional relationships of the related HDAC-1 corepressors during embryogenesis. First, we determined whether *hda-1*, *let-418*, *sin-3*, and *spr-1* participate in embryogenesis. Then, we analyzed the genetic interactions between two corepressors to identify relationships among all three corepressors. Finally, comprehensive comparative analysis of the target genes of the LET-418, SIN-3, and SPR-1 complexes was performed via RNA sequencing (RNA-seq). We then combined our analysis of genetic interactions with gene ontology (GO) analysis of these corepressors, which suggests a prominent overlapping role among SIN-3, SPR-1, and LET-418 and between SIN-3 and SPR-1.

## Materials and methods

### *Caenorhabditis elegans* strains

*Caenorhabditis elegans* strains were derived from the wild-type (WT) Bristol strain (Brenner 1974). Worms were incubated on nematode growth medium (NGM) and fed OP50 bacteria at 20 °C. When performing RNA-interference (RNAi) experiments, the animals were fed dsRNA-expressing *Escherichia coli* HT115 (DE3), which were maintained at 20 °C.

*Caenorhabditis elegans* strains with the following putative null alleles were used for our analysis: *sin-3(tm1276*) (National BioResource Project, Japan), *spr-1(ok2144)* (*C. elegans* Gene Knockout Consortium), and weak loss-of-function allele *let-418 (n3536)* (Ceol et al. 2006) (Caenorhabditis Genetics Center). To compare the gene functions of these three corepressors under the same conditions, we analyzed *let-418(n3536)* in a semi-permissive condition at 20 °C.

### Sample preparation for RNA-seq

To isolate synchronized early *C. elegans* embryos, the following four steps were performed. (1) Adult worms (WT and mutant) bearing fertilized eggs were treated with bleach solution, and the eggs were extracted. (2) The eggs were cultured in S-basal until all eggs hatched to synchronize the developmental stage, and subsequently OP50 solution was added to the S-basal. (3) The synchronized worms were incubated until they grew to the young adult stage, capable of bearing 2–3 fertilized eggs. (4) The early embryos were isolated by bleaching.

### Total RNA extraction

For RNA-seq and reverse-transcriptase quantitative polymerase chain reaction (RT-qPCR) analyses, total RNA was extracted from the WT, *let-418(n3536)*, *sin-3(tm1276)*, and *spr-1(ok2144)* strains using the TRI Reagent (Molecular Research Center, Inc., Cincinnati, OH). Following DNA digestion, total RNA was extracted using an RNeasy Mini Kit (Qiagen, Hilden). The extracted RNA was qualitatively evaluated using a Bioanalyzer (Agilent Technologies, Palo Alto, CA) and the Agilent RNA 6000 Nano Kit (Agilent Technologies, Palo Alto, CA).

### RT-qPCR analysis

Complementary DNA (cDNA) was synthesized from total RNA from WT *C. elegans* at each developmental stage (early-stage embryo, middle-stage embryo, late-stage embryo, first larva, and young adult) using the PrimeScript RT Reagent Kit (Takara, Kusatsu). RT-qPCR was performed in a StepOnePlus™ qPCR system (Thermo Fisher Scientific, Waltham, MA) using THUNDERBIRD SYBR qPCR Mix (Toyobo, Osaka). The expression levels of *hda-1*, *sin-3*, *let-418*, and *spr-1* were normalized to those of a gene encoding an iron binding protein (Y45F10D.4), which was previously characterized as a reference gene because its expression is stable during development in both WT and mutant strains (Hoogewijs et al. 2008). The following primers were used to amplify *Y45F10D.1* (Y45F10D.4_F, 5′-GTCGCTTCAAATCAGTTCAGC-3′; Y45F10D.4_R, 5′-GTTCTTGTCAAGTGATCCGACA-3′), *hda-1* (hda-1_F, 5′-GGTCAAGGGCACGTCATGAAGCC-3′; hda-1_R, 5′-CTCGTCGCTGTGAAAACGAGTC-3′), *let-418* (let-418_F, 5′-GTGCTGCTATCGGATTGACAGACG-3′; let-418_R, 5′-GGGTTTGCCTCCAGTATTTGTGGC-3′), *sin-3* (sin-3_F, 5′-GCAACCGTGGAATTGATGA-3′; sin-3_R, 5′-GTTGATTCGGTGTTGTTCGAC-3′), and *spr-1* (spr-1_F, 5′-CTCCATCTCCATATCCTGAAGC-3′; spr-1_R, 5′-GCACGGCATTCTGGACGATTCATCG-3′).

### Feeding RNAi

RNAi was performed using the feeding RNAi method with freshly prepared RNAi feeding plates, as described previously (Kamath et al. 2001). Full-length *hda-1*, *let-418*, *sin-3*, and *spr-1* cDNA was isolated from a *C. elegans* cDNA library and inserted into the feeding RNAi vector L4440 (Addgene, Cambridge, MA, USA). An L4440 vector lacking an insert was used as a negative control. After confirming that each inserted sequence was correct, the feeding vectors were individually transformed into *E. coli* HT115(DE3) cells, which were then seeded on NGM agar plates containing Luria–Bertani medium and 50 μg/mL ampicillin and cultured for 12 h. Then, each culture was seeded onto a 60 mm NGM agar feeding plate containing 50 µg/mL ampicillin and 1 mM isopropyl β-d-1-thiogalactopyranoside and then incubated at 25 °C for 8 h. L4-stage worms were transferred onto a feeding plate and cultured at 20 °C. The phenotypes of the F2 embryos were analyzed, except for those fed RNAi bacteria expressing double-stranded *hda-1* RNA, which were analyzed in the F1 embryos.

### Analysis of embryonic lethality

To analyze embryonic lethality, fertilized eggs were isolated by dissecting 1-day-old adult worms, after which the fertilized eggs were incubated at 20 °C for 24 h and the ratio of the unhatched embryos was scored. To characterize the timing of the terminal phenotype, we defined embryonic lethality in early embryos as those that died before the ventral cleft-enclosure stage. Embryonic lethality in middle embryos was defined as those that died between the ventral cleft-enclosure stage and the comma stage. Embryonic lethality in late embryos was defined as those that died between the 1.5-fold stage and the threefold stage. To analyze the terminal phenotypes of the dead embryos, Nomarski microscopy was performed using a Zeiss Axio Imager A1 microscope equipped with an EC Plan-Neofluar 40 × NA, 0.75 objective (Zeiss), AxioVision software (Zeiss), and an AxioCam MRc digital camera. The images were processed using Adobe Photoshop CS6.

### Statistical analyses of embryonic lethality

*P*-values (determined using Fisher’s exact test) were used to assess the significance of differences observed in terms of embryonic lethality. To analyze the embryonic lethality of *let-418(n3536);control(RNAi)*, *let-418(n3536);sin-3(RNAi)*, *let-418(n3536);spr-1(RNAi)*, *sin-3(tm1276);control(RNAi)*, *sin-3(tm1276);let-418(RNAi)*, *sin-3(tm1276);spr-1(RNAi), spr-1(ok2114);control(RNAi)*, *spr-1(ok2114);let-418(RNAi)*, and *spr-1(ok2114);sin-3(RNAi)* strains, the numbers of embryonic-lethal embryos and hatched (non-embryonic-lethal) embryos were compared.

### RNA-seq analysis

RNA-seq analysis (N = 3) of the WT, *sin-3(tm1276)*, *let-418(n3536)*, and *spr-1(ok2144)* strains was performed using a MiSeq instrument (Illumina, San Diego, CA), following the manufacturer’s recommended protocols (available on the Illumina website). Library preparation for RNA-seq was performed using the TruSeq Stranded Total RNA LT Sample Prep Kit (Illumina, San Diego, CA). Next, the sample DNA was denatured using a MiSeq Reagent Kit v3 (Illumina, San Diego, CA), diluted, and subjected to paired-end sequencing (75 base pairs) in a MiSeq instrument (Illumina, San Diego, CA). Although the RNA-seq analysis of the *sin-3* mutant has been reported previously (Beurton et al. 2019), we analyzed the gene expression profile of this mutant strain to compare the gene expression between the WT and the three corepressor mutants under the same conditions.

### RNA-seq data analysis

The quality of raw sequence data obtained by RNA-seq was checked using FastQC software. Trimmomatic software (Bolger et al. 2014) was employed to trim low-quality reads, and the sequence data were mapped to a *C. elegans* reference genome (WormBase Version 261) using HISAT2 software. The count data of WT and mutants were compared using DESeq2 software (Love et al. 2014) and differentially expressed genes (DEGs; *p* value < 0.01, log_2_ fold-change; positive or negative) were identified according to a previously described method (Nomoto et al. 2019). To further analyze the DEGs, we identified upregulated genes (log_2_ fold-change > 1 and *p* value < 0.01) and downregulated genes (log_2_ fold-change < − 1 and *p* value < 0.01). Using the DAVID Bioinformatics Resource database (version 6.8) (Dennis et al. 2003), GO enrichment analyses were performed to identify the specific functions of the DEGs.

## Results

### *hda-1*, *sin-3*, *let-418*, and *spr-1* play a role in *C. elegans* embryogenesis

**Fig. 1:**
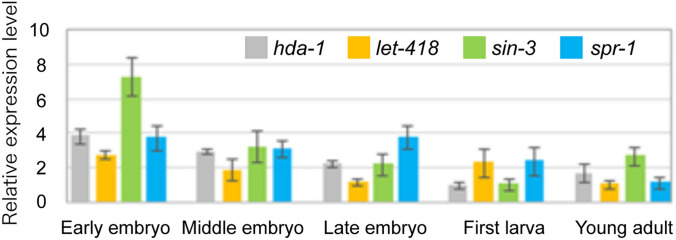
Comparison of the relative mRNA expression levels of *hda-1*, *let-418, sin-3*, and *spr-1* genes during development. The mRNA expression levels of *hda-1*, *let-418*, *sin-3*, and *spr-1* in the early-embryo, middle-embryo, late-embryo, first-larva, and young-adult stages in WT worms were analyzed by RT-qPCR (N = 3). The Y45F10D.4 (iron binding protein) gene was used as a reference. The bars and error bars indicate the relative mRNA expression levels and standard deviations, respectively, of *hda-1* (gray), *let-418* (orange), *sin-3* (green), and *spr-1* (light blue) at each developmental stage (color figure online)

The activities of *hda-1*, *sin-3*, *let-418*, and *spr-1* during embryonic development in *C. elegans* were confirmed through determining relative mRNA expression levels and embryonic lethality after either gene knockdown or deletion. Changes in the relative mRNA expression levels of *hda-1*, *let-418, sin-3*, and *spr-1* in *C. elegans* during development were analyzed by RT-qPCR at five developmental stages—the early-embryo, middle-embryo, late-embryo, first-larval, and young adult stages. We found that all analyzed genes were expressed throughout embryonic development (Fig. 1). Our results showed that *hda-1* expression started at the early embryonic stage in *C. elegans* is consistent with previous results obtained with *C. elegans*, zebrafish, and mice (Dufourcq et al. 2002; Ma and Schultz 2016; Pillai et al. 2004).

**Fig. 2:**
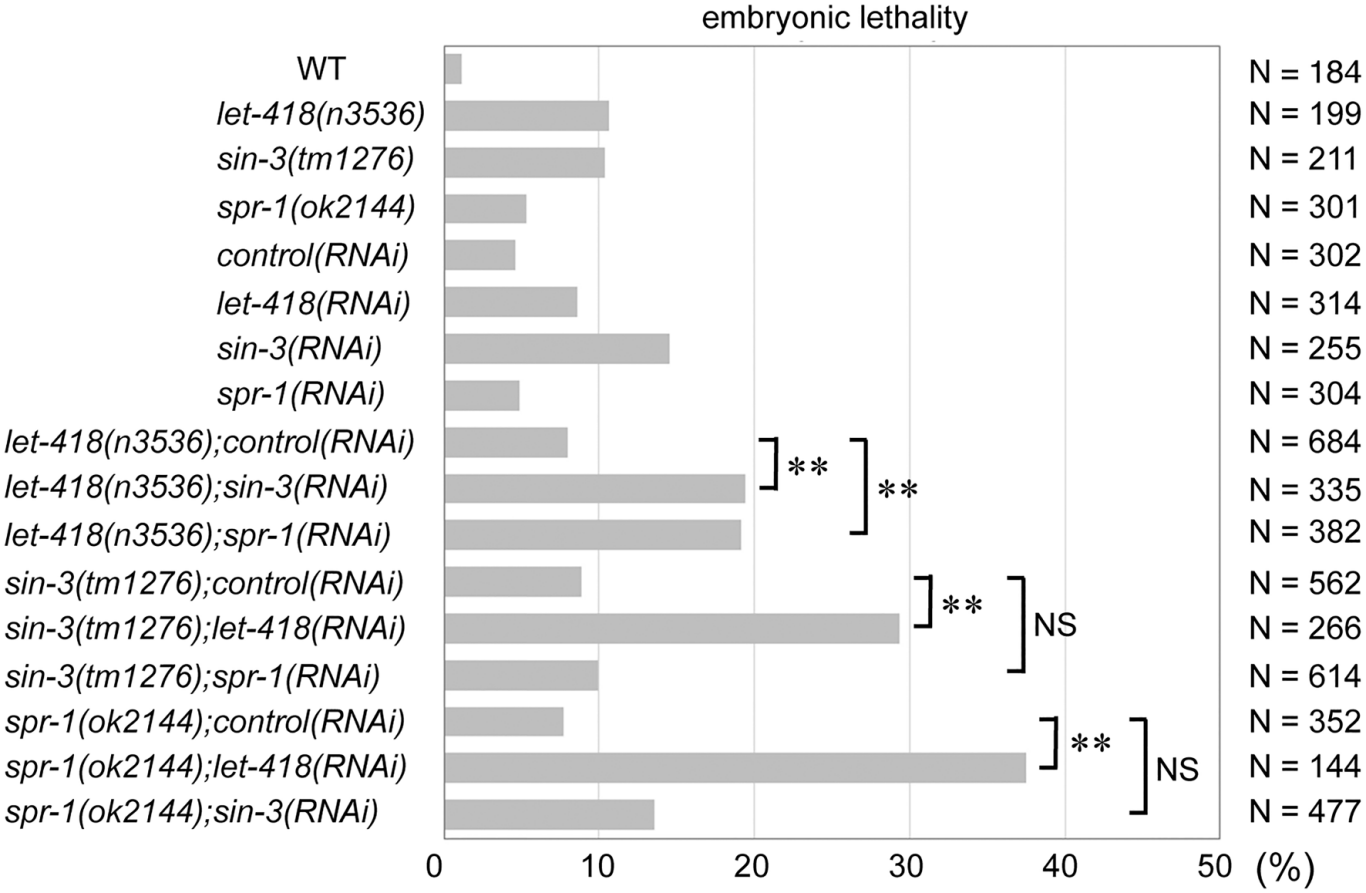
Genetic interactions among the *sin-3*, *let-418*, and *spr-1* mutants. Embryonic lethality of the WT, *let-418(n3536), sin-3(tm1276)*, and *spr-1(ok2144)* strains (single and double mutants) was analyzed by performing RNAi-based knockdown analysis. Each gray box indicates the embryonic lethality of the WT strain and the indicated mutant. *p* values are indicated for Fisher’s exact test comparisons with *sin-3(tm1276);control(RNAi)*, *let-418(n3536);control(RNAi)*, or *spr-1(ok2144);control(RNAi)*. **p value < 0.01. NS, not significant. N, number of embryos observed

Because *hda-1(e1795)* mutants are completely sterile (Dufourcq et al. 2002), we used *hda-1* feeding RNAi to analyze the function of this gene during embryonic development. During *C. elegans* embryogenesis, the effects of HDA-1 and its corepressors were analyzed by observing the embryonic lethality of *hda-1(RNAi)*, *sin-3(tm1276)* deletion, *spr-1(ok2144)* deletion, and *let-418(n3536)* temperature-sensitive weak allele. The results show that the embryonic lethality of the *let-418(n3536)*, *sin-3(tm1276)*, and *spr-1(ok2144)* mutants (10.6%, 10.4%, and 5.3%, respectively) was much higher than that of the WT strain (1.1%) (Fig. 2). Similarly, *hda-1(RNAi)* showed higher embryonic lethality (99.7%, N = 352; data not shown) compared to the control (*RNAi*) (4.6%) (Fig. 2). The embryonic lethality of *hda-1(RNAi)* and *sin-3(tm1276)* mutants was consistent with the findings of previous reports, whereas the embryonic lethality of the *let-418(n3536)* strain under semi-permissive conditions (maintained at 20 °C) was inconsistent (Beurton et al. 2019; Shi and Mello 1998; Turcotte et al. 2018). Overall, relative mRNA expression levels and embryonic lethality rates indicate that *hda-1*, *sin-3*, *let-418*, and *spr-1* play a role in *C. elegans* embryogenesis.

**Fig. 3:**
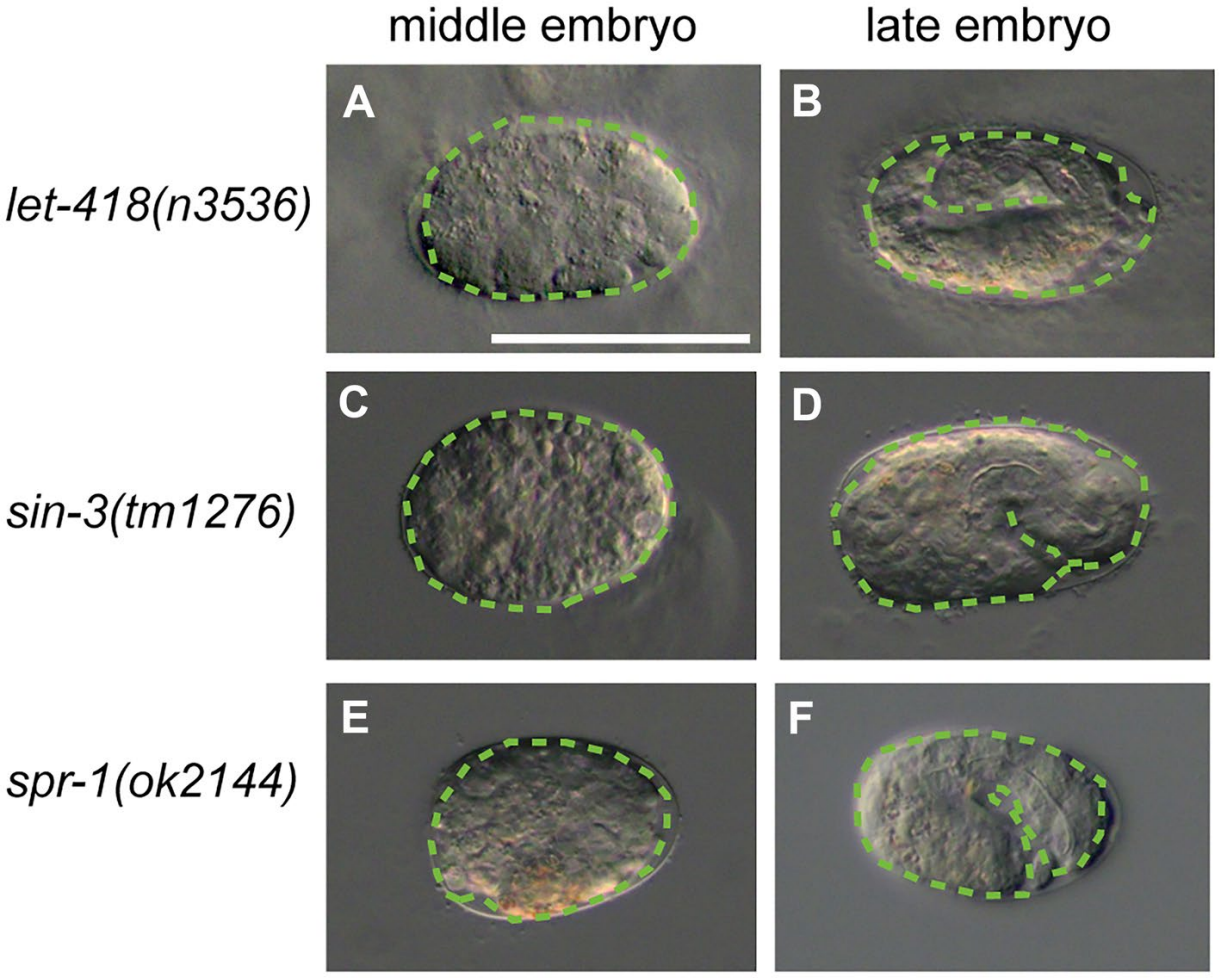
Microscopic images of the terminal phenotypes of *let-418*, *sin-3*, and *spr-1* mutants with embryonic lethality. Differential-interference contrast micrographs of the *let-418(n3536)* (**a**, **b**), *sin-3(tm1276)* (**c**, **d**), and *spr-1(ok2144)* (**e**, **f**) mutants. **a**–**f** The middle-stage (**a**, **c**, **e**) and late-stage (**b**, **d**, **f**) embryos that exhibited a terminal phenotype of embryonic lethality are indicated. The green dotted lines outline the embryos in each panel. White scale bar, 50 μm (color figure online)

Additionally, we observed the terminal phenotypes of knockdown and mutant embryos using a differential-interference contrast microscope. Similar to that in *hda-1(RNAi)* embryos, which was described previously (Shi and Mello 1998), the development of most embryonic-lethal embryos stopped between the ventral cleft-enclosure stage and the threefold stage in the *let-418(n3536)*, *sin-3(tm1276*), and *spr-1(ok2144)* mutants (Fig. 3, Table 1). These results indicate that each corepressor is crucial for progression to the middle- and late embryonic developmental stages of *C. elegans*. However, the developmental timing of the embryonic lethality showed no difference. Thus, we could not check the epistatic relationship among the three corepressor components because of the similar terminal phenotypes results.

**Table 1:** Timing of embryonic lethality in *let-418, sin-3*, and *spr-1* mutants

	Before ventral cleft enclosure (Early embryo)	Ventral cleft enclosure to comma (Middle embryo)	1.5-fold to 3-fold (Late embryo)	Total number of embryos
*let-418(n3536)*	1 (2.4%)	25 (61.0%)	15 (36.6%)	41
*sin-3(tm1276)*	2 (4.0%)	36 (72.0%)	12 (24.0%)	50
*spr-1(ok2144)*	0 (0%)	23 (65.7%)	12 (34.3%)	35

### Genetic interactions among *let-418*, *sin-3*, and *spr-1* during embryonic development suggest possible overlapping roles among these genes

To identify the functional relationships among *sin-3*, *spr-1*, and *let-418*, we analyzed the genetic interactions among these corepressors by examining the rates of embryonic lethality in strains containing an RNAi-mediated knockdown of a specific gene in a distinct gene deletion background. In an *spr-1(ok2144)* deletion background and a *sin-3(tm1276)* deletion background, the embryonic lethality of the *sin-3(tm1276*)*;spr-1(RNAi)* and *spr-1(ok2144);sin-3(RNAi)* mutants (9.9% and 13.6%, respectively) was comparable to that of *sin-3(tm1276);control(RNAi)* and *spr-1(ok2144);control(RNAi)* strains (8.9% and 7.7%, respectively). These results suggest that *sin-3* and *spr-1* may have prominent overlapping roles in *C. elegans* during embryogenesis. In contrast, the embryonic lethality of the *sin-3(tm1276*)*;let-418(RNAi)* and *spr-1(ok2144);let-418(RNAi)* mutants (29.3% and 37.5%, respectively) was significantly higher than that of *sin-3(tm1276);control(RNAi)* and *spr-1(ok2144);control(RNAi)* (8.9% and 7.7%, respectively) (Fig. 2). These results suggest that *let-418* has a prominent specific role distinct from that of either *sin-3* or *spr-1*. To further evaluate a possible functional relationship among *sin-3*, *spr-1*, and *let-418*, we also examined the rate of embryonic lethality in strains containing RNAi-mediated knockdown of *sin-3* and *spr-1* in a *let-418(n3536)* weak allele background. The embryonic lethality of the *let-418(n3536);sin-3(RNAi)* and *let-418(n3536);spr-1(RNAi)* mutants (19.4% and 19.1%, respectively) was significantly higher than that of *let-418(n3536);control(RNAi)* (8.0%); these results also suggest that *let-418* has a prominent specific role distinct from that of either *sin-3* or *spr-1* (Fig. 2). In contrast, the comparable embryonic lethality results of the *let-418(n3536)*; *sin-3(RNAi)* and *let-418(n3536)*; *spr-1(RNAi)* mutants may also indicate a possible overlapping role among these three genes. Taken together, our genetic interaction analyses suggest a prominent overlapping role among *sin-3*, *spr-1*, and *let-418* and between *sin-3* and *spr-1*, and a prominent exclusive role of *let-418* compared to that of either *sin-3* or *spr-1*.

### Analysis of transcriptionally regulated genes in let-418, sin-3, and spr-1 mutants

**Fig. 4:**
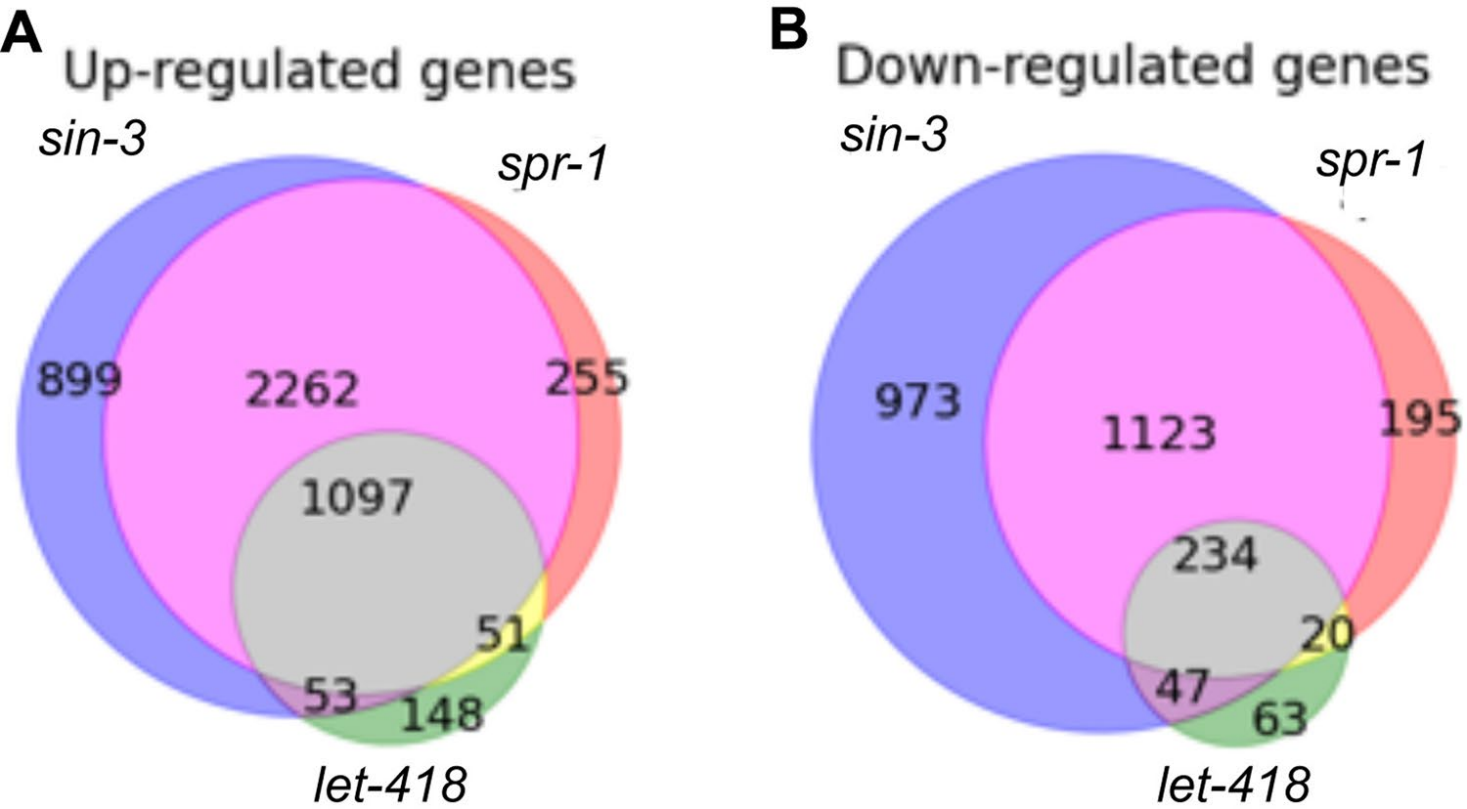
Comparison of mRNA expression levels in corepressor mutants by RNA-seq. Venn diagrams showing overlapping upregulated genes (**a**) and downregulated genes (**b**) in *let-418* embryos (green), *sin-3* embryos (blue), *spr-1* embryos (red), *let-418* and *sin-3* embryos (purple), *let-418* and *spr-1* embryos (yellow), *sin-3* and *spr-1* embryos (magenta) and *let-418*, *sin-3*, and *spr-1* embryos (gray) (color figure online)

To identify genes that were transcriptionally regulated by the three HDAC-1 complexes, we identified groups of genes for which expression levels significantly fluctuated among the corepressor mutants (Fig. 4, Supplementary Fig. 1–3, Supplementary Table 1). Expression-level information for 46,756 transcripts in early WT, *let-418(n3536)*, *sin-3(tm1276)*, and *spr-1(ok2144)* mutant (N = 3) embryos was obtained by performing RNA-seq analysis. Differentially expressed genes (DEGs) in each mutant strain compared to those in the WT were defined as those for which expression levels were significantly upregulated (*p* value < 0.01 and log_2_ fold-change > 1) or significantly downregulated (*p* value < 0.01 and log_2_ fold-change < 1) (Fig. 4). Genes for which expression levels were significantly upregulated or downregulated in the corepressor mutants were defined as transcriptionally repressed and promoted genes, respectively (Supplementary Table. 2).

The transcriptionally repressed genes showed noticeably higher expression levels than the promoted genes in all DEGs results of corepressors, which is consistent with the role of HDAC complexes in negatively regulated transcription. A Venn diagram of the transcriptionally repressed and promoted genes among corepressors showed a similar pattern. Consistent with our observations in genetic interactions analyses, *sin-3* and *spr-1* showed highly overlapped targets in both transcriptionally repressed and promoted genes. The overlapped targets between *sin-3, spr-1*, and *let-418* were also high, both in the transcriptionally repressed and promoted genes, but less common than the *sin-3* and *spr-1* shared targets; this indicates that the related repressor complexes of each of the three corepressor components may cooperatively regulate some expression levels or may have compensatory relationships during *C. elegans* embryogenesis. Considering the utilization of standard bulk RNA-seq analysis in this study, another possibility is that the related repressor complexes of the three corepressor components may have some similar roles, but each repressor complex independently plays the role within a specific cell.

The DEGs were not consistent with our genetic interactions analyses for *let-418* specific targets. The DEG results showed that expression of the *let-418* specific targets was noticeably lower, indicating an implausible single prominent role compared to either *sin-3* or *spr-1*. Basically, the fluctuated expression levels recorded in *let-418* mutants were less frequent compared to those of either *sin-3* or *spr-1* mutants. Indeed, usage of the weak loss-of-function *let-418* mutants was likely a major factor for the less prominent than expected RNA-seq results and might overshadow the actual outcomes. However, the results from this *let-418* mutant may still reflect the genes strongly related to *let-418*. Overall, the DEGs results indicate the occurrence of prominent shared roles among *sin-3, spr-1*, and *let-418* and between *sin-3 and spr-1,* of which the fluctuated genes groups were determined as LET-418–SIN-3–SPR-1 and SIN-3–SPR-1, respectively.

### GO enrichment analysis of transcriptionally regulated genes in the LET-418–SIN-3–SPR-1, SIN-3–SPR-1, and LET-418 pathways

As shown above, our genetic interaction and DEG analyses agree on the shared roles between *sin-3* and *spr-1* and among *sin-3, spr-1*, and *let-418*, but were inconsistent regarding the *let-418* specific role. Genetic interactions indicate that *let-418* may have prominent unique functions, whereas DEG analysis identified genes with low, fluctuating expression levels that are specific to *let-418*. To confirm these, we conducted GO enrichment analysis in both transcriptionally repressed and promoted genes for the fluctuating genes groups in LET-418–SIN-3–SPR-1, SIN-3–SPR-1, and the fluctuating genes from *let-418(n3536)* that did not overlap with other corepressors, determined as LET-418 (Fig. 4, Supplementary Table.3). We highlighted the GO enrichment results related to embryogenic development, cell specification, cell differentiation, cellular function, gene expression, and molecular function. We focused on differences and similarities in GO terms to gain further insight into the overlapping and non-overlapping roles among corepressors in *C. elegans* embryogenesis.

**Fig. 5:**
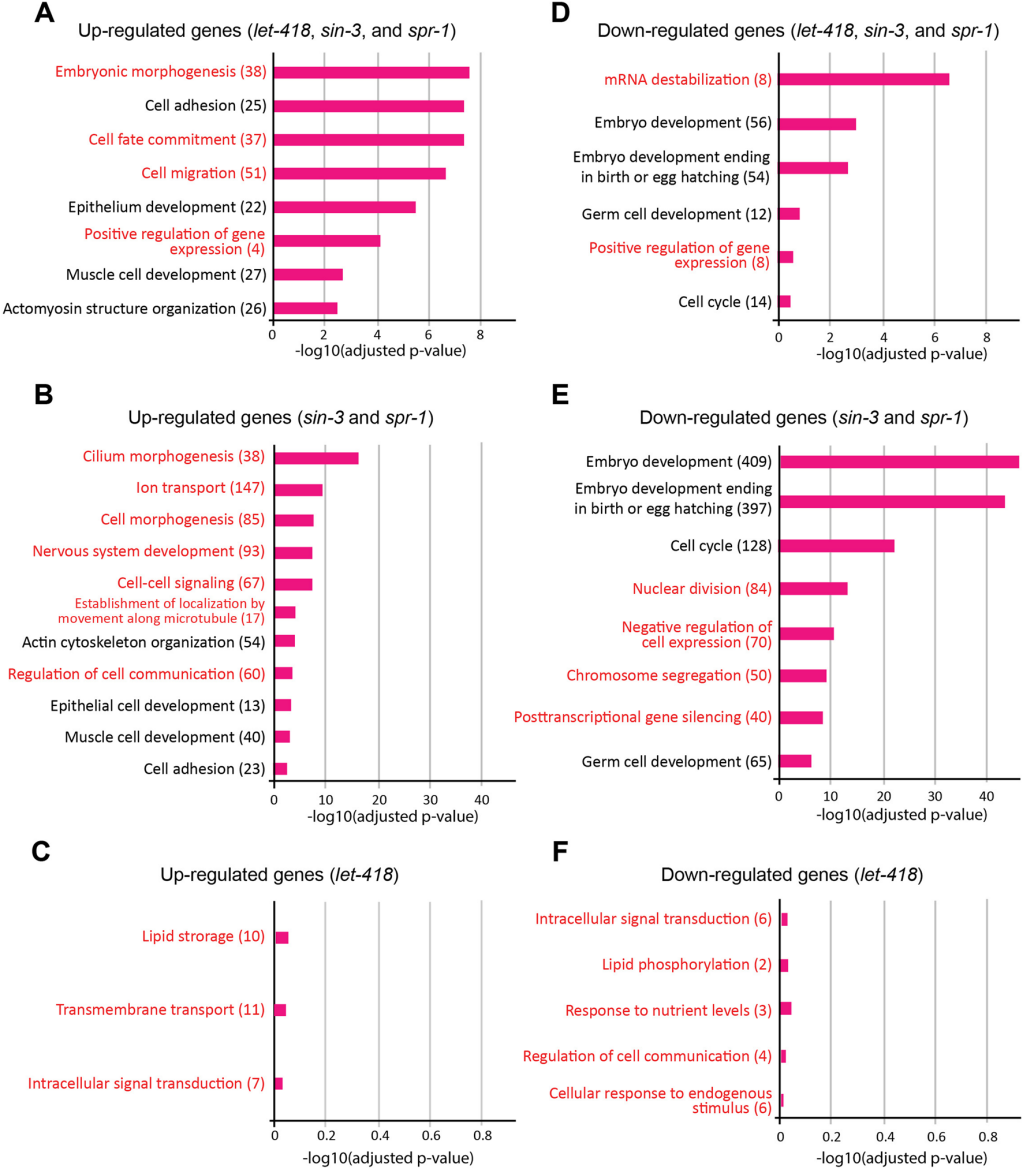
Gene ontology (GO) analysis of genes dysregulated by the LET-418–SIN-3–SPR-1, SIN-3–SPR-1, and LET-418 pathways. GO terms of the gene groups that were elucidated based on upregulated genes (**a**–**c**) and downregulated genes (**d**–**f**) from the corepressor mutants are indicated. **a** GO terms associated with genes that were transcriptionally repressed by the LET-418–SIN-3–SPR-1 pathway. **b** GO terms associated with genes that were transcriptionally repressed by the SIN-3–SPR-1 pathway. **c** GO terms associated with genes that were transcriptionally repressed by the LET-418 pathway. **d** GO terms associated with genes that were transcriptionally promoted by the LET-418–SIN-3–SPR-1 pathway. **e**)GO terms associated with genes that were transcriptionally promoted by the SIN-3–SPR-1 pathway. **f** GO terms associated with genes that were transcriptionally promoted by the LET-418 pathway. The terms indicated with black text were identified as common GO terms between the LET-418–SIN-3–SPR-1 pathway and the SIN-3–SPR-1 pathway. The red text indicates specific GO terms associated with the LET-418–SIN-3–SPR-1 pathway, the SIN-3–SPR-1 pathway, and the LET-418 pathway. The *p* values of the GO terms were determined to evaluate the potential relevance of the associated biological pathways. The numbers in each set of parentheses indicate the numbers of genes that were associated with each GO term (color figure online)

The resulting upregulated genes from each compressor mutant may be directly or indirectly associated with negative gene regulation role of each corepressor. The GO enrichment results of LET-418–SIN-3–SPR-1 clearly indicate that the interplay among *sin-3, spr-1,* and *let-418* may be essential to negatively regulate embryonic morphogenesis, cell fate commitment, and positive regulation of gene expression (Fig. 5). The interplay among *sin-3, spr-1,* and *let-418* is also significantly related to cell adhesion, epithelium/epithelial and muscle cells development, and actin cytoskeleton organization, but without *let-418,* the interplay among *sin-3 and spr-1* is still able to regulate these biological processes. In contrast, SIN-3–SPR-1 is specifically associated with numerous biological processes such as cilium morphogenesis, ion transport, cell morphogenesis, nervous system development, cell–cell signaling, establishment of localization along microtubule, and regulation of cell communication (Fig. 5). Although the upregulated genes of LET-418 were associated with GO terms such as lipid storage, transmembrane transport, and intracellular signal transduction, the *p*-values did not reflect statistically significant differences (Fig. 5).

Because the corepressors are mainly associated with the negative regulation of gene expression, the downregulated genes resulting from either corepressor mutant are supposed to be transcriptionally promoted genes caused by repressing activity on other genes (indirectly promoted from repressing activity on other targets). The GO enrichment in the downregulated genes from LET-418–SIN-3–SPR-1 group yielded unique biological processes such as mRNA destabilization and positive regulation of gene expression. Additionally, the GO enrichment in LET-418–SIN-3–SPR-1 had similar results with SIN-3–SPR-1 on embryo development, embryo development ending in birth or egg hatching, germ cell development, and cell cycle, indicating that the interplay between *sin-3* and *spr-1* with *let-418* in these biological processes may be compensated in the absence of *let-418*. In contrast, the downregulated genes of SIN-3–SPR-1 were significantly associated with nuclear division, negative regulation of gene expression, chromosome segregation, and posttranscriptional gene silencing. Although genes specifically downregulated in the *let-418* mutant were associated with GO terms such as cellular response to endogenous stimulus, intracellular signal transduction, response to nutrient levels, lipid phosphorylation, and regulation of cell communication, the *p-*values did not indicate that these associations were statistically significant (Fig. 5). These results imply that relatively few transcripts in *let-418(n3536)* are affected at a semi-permissive temperature.

We also identified the genes that were significantly upregulated and downregulated in a single mutant of either *sin-3, spr-1,* and *let-418* and related to embryogenesis based on the previous GO enrichment results (See Supplementary Fig. 1–3 for the 10 most significantly upregulated and downregulated genes). ECM-related genes were among the most upregulated genes in all three mutants. Genes encoding extracellular matrix (ECM) components, *noah-1*, *lam-2*, and *lam-3*, an ECM receptor, *dgn-1*, and a putative matrix proteinase inhibitor, *mig-6*, were among the most significantly upregulated genes in the *let-418(n3536)* mutant. In *sin-3*(*tm1276*), three ECM genes, *noah-1*, *lam-3*, and *nid-1*, as well as *mig-6*, were significantly upregulated. In addition, three ECM genes, *noah-2*, *lam-3*, and *nid-1*, and *mig-6* were upregulated in the *spr-1(ok2144)* mutant*.* In contrast to the upregulated genes, we did not identify any similarly downregulated genes among the three mutants. These results indicate that all three class-I HDAC-1 corepressors significantly repress the expression of ECM-related genes.

## Discussion

During embryogenesis, cells actively undergo division and differentiation by following highly regulated genetic and epigenetic mechanisms. HDAC, a class of epigenetic regulators, catalyzes heterochromatin formation on specific genomic regions by removing acetyl groups from histone tails that results in transcriptional inhibition. The two members of Class-I HDAC, HDAC-1 and -2, are essential for accurate cell division and the pluripotency of embryonic stem (ES) cells. However, only HDAC-1 is essential for controlling ES cell differentiation (Dovey et al. 2010; Jamaladdin et al. 2014). In *C. elegans*, HDAC-1 is able to form a complex with three distinct corepressor components, SIN-3, SPR-1, and LET-418. HDAC-1 forms the Sin3 and CoREST complexes while interacting with SIN-3 and SPR-1, respectively (Hayakawa and Nakayama 2011). Furthermore, HDAC1 interaction with LET-418 can form two distinct complexes, MEC and NuRD, depending on the presence of the other components (Passannante et al. 2010). Here, we initially confirmed that *hda-1*, *sin-3*, *spr-1*, and *let-418* play a role during embryogenesis in *C. elegans* through mRNA expression, embryonic lethality given either gene knockdown or deletion, and terminal phenotype analyses. Further, our terminal phenotype observations demonstrate that either gene is similarly crucial during the middle- and late embryonic developmental stages. The similar terminal phenotype results, however, complicate the determination of epistatic relationships among these corepressor components. Further studies are required to identify the signal transduction cascades activated by these corepressors in each pathway.

Although HDAC-1 is known to be essential protein during embryogenesis, the functional relationships among its complexes remain unclear. Here, we sought to elucidate those through genetic interactions and DEG analyses. Our results suggest prominent shared roles between *sin-3* and *spr-1* and among *sin-3, spr-1*, and *let-418*. In contrast, genetic interactions indicate *let-418* as having a prominent independent role, whereas DEG analysis of the *let-418* mutant identified only genes with low, fluctuating gene expression that did not overlap with either *sin-3* or *spr-1* (Fig. 4). A possible explanation for these results is that the repressor complex involving LET-418 may be regulated by another repressor complex or may frequently function dependent on another repressor complex. However, it should be noted that the *let-418* mutant used in this study is a weak allele of *let-418*, and, therefore, the transcriptome analysis may not fully reflect the normal function of this gene during embryonic development.

**Fig. 6:**
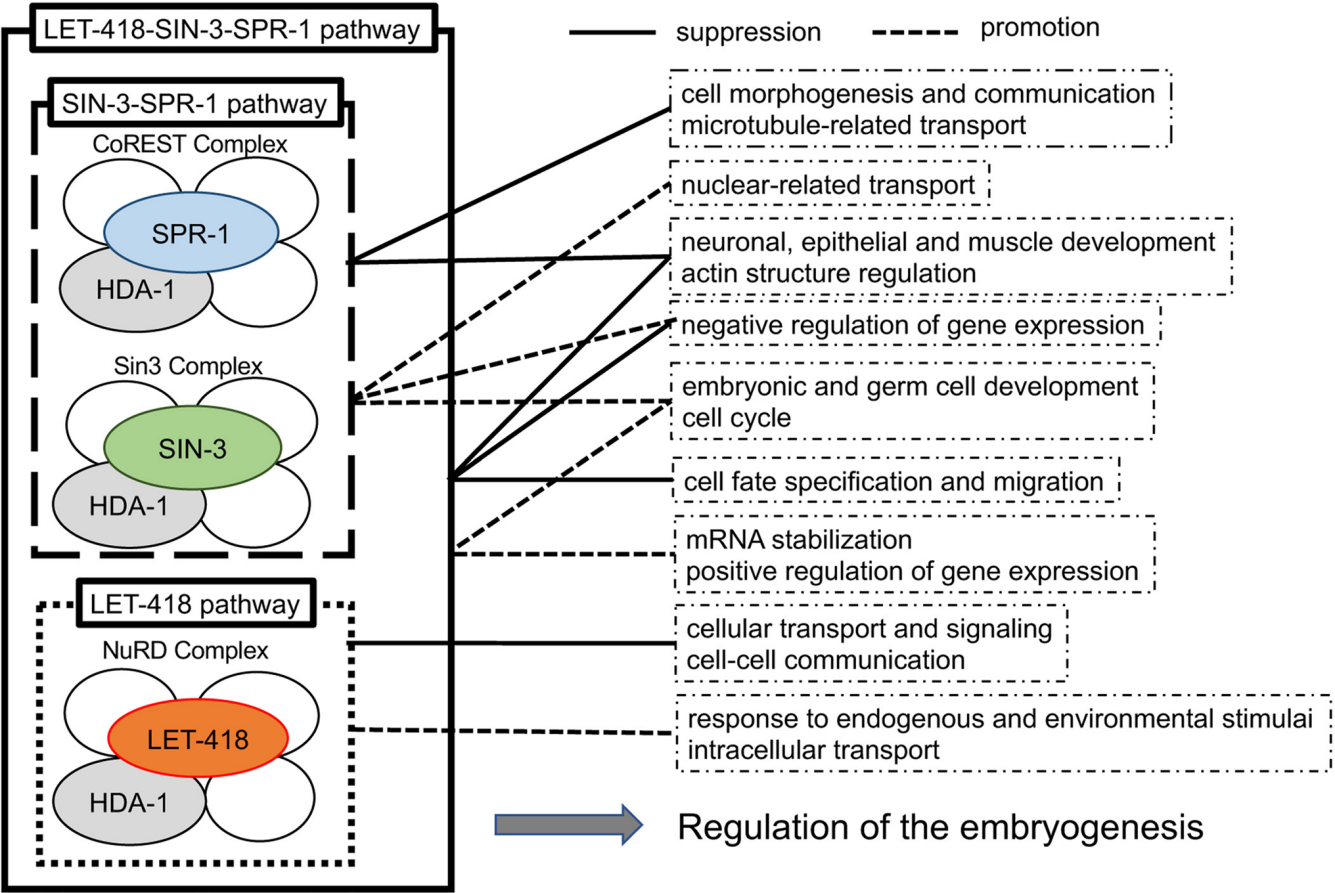
Model of the functional relationships among the LET-418–SIN-3–SPR-1, SIN-3–SPR-1, and LET-418 pathways. The LET-418–SIN-3–SPR-1, SIN-3–SPR-1, and LET-418 pathways positively and negatively regulate common and pathway-specific biological functions to influence embryonic development

Comparative GO analysis of the fluctuated genes among the three specified groups, LET-418–SIN-3–SPR-1, SIN-3–SPR-1, and LET-418, indicates that similar GO terms were enriched between the LET-418–SIN-3–SPR-1 group and the SIN-3–SPR-1 group but not the LET-418 group. Further analyses of the GO terms indicate that many of the suppressed genes are related to neuronal, epithelial, and muscle development and actin-structure regulation. Enhanced expression of genes related to embryonic and germ cell development and cell cycle progression was identified as a common feature between the fluctuated genes in LET-418–SIN-3–SPR-1 and SIN-3–SPR-1 groups (Fig. 6). There are several possibilities that can explain the overlapping fluctuating genes either in LET-418–SIN-3–SPR-1 group or SIN-3–SPR-1 group. First, the related repressor complexes may cooperatively regulate some expression levels or may have compensatory relationships. This possibility implies that the cooperative regulation of gene expression among the related repressor complex is important for the precise regulation during embryonic development. Second, the related repressor complexes may have similar functions but each repressor complex independently plays a role within a specific cell. Further investigation is needed to clarify this issue.

GO enrichment analysis of the repressed genes in the SIN-3–SPR-1 group indicates that they are related to cell morphogenesis, intracellular communication, and microtubule-related transport (Fig. 6). In contrast, the promoted genes of this group are related to nuclear-related cell division and negative regulation of gene expression (Fig. 6). How the SIN-3-SPR-1 group regulates these biological functions? Interestingly, mSin3A and CoREST are co-expressed in mouse embryos at E11.5, and mSin3A has been shown to act as a functional component of the REST–CoREST suppressor complex (Grimes et al. 2000). Thus, negative transcriptional regulation of the SIN-3–CoREST suppressor complex may be conserved in both vertebrates and invertebrates.

GO enrichment analysis indicates that the repressed genes in LET-418–SIN-3–SPR-1 group are related to cell morphogenesis, cell fate specification, cell migration, and negative regulation of gene expression (Fig. 6). Although *hda-1* has been shown to positively regulate neuronal and distal-tip cell migration during post-embryonic development in *C. elegans* (Dufourcq et al. 2002; Zinovyeva et al. 2006), here we show that the related repressor complexes of LET-418, SIN-3, SPR-1 likely modulate the negative regulation of cell migration during embryonic development. In contrast, the promoted genes in LET-418–SIN-3–SPR-1 group are related to mRNA stabilization and positive regulation of gene expression. Differences in gene expression and mRNA stabilization occur in somatic and germ cell linages throughout embryonic development in *C. elegans*, which might reflect the function of LET-418, SIN-3, and SPR-1. Taken together, our results indicate that the three corepressor components positively and negatively regulate cell type-specific functions during embryogenesis. However, the exact mechanism of the LET-418-, SIN-3-, and SPR-1-related repressor complexes in negatively regulating cell differentiation and movement, promoting cell type-specific gene stabilization, and positively and negatively regulating the expression levels of different genes remains unknown.

The repressed genes in the LET-418-specific group are related to the control of cellular transport and signaling. In contrast, the promoted genes are related to controlling cell–cell communication, cellular responses to endogenous and environmental stimuli, and intracellular transport (Fig. 6). Our results are inconsistent with previous studies on LET-418 function during embryogenesis, which are related to the prevention of germline development and repressing the neuronal fate during embryonic development (Käser-Pébernard et al. 2016; Unhavaithaya et al. 2002). We used a weak allele of the *let-418* mutant, and further studies with a strong loss-of-function *let-418* mutant are required to confirm its normal cellular roles.

We also highlighted several mostly fluctuating genes in DEG analyses (Supplementary Fig. 1–3). Similar to previous findings indicating that ECM genes are upregulated in *hda-1(RNAi)* embryos (Whetstine et al. 2005), we found that genes encoding ECM- and ECM-related mRNAs are significantly upregulated in the *C. elegans* HDAC-1 corepressor mutants, specifically *let-418(n3536)*, sin-3*(tm1276)*, and *spr-1(ok2144)*. The upregulated ECM genes (*noah-1*, *noah-2, nid-1*, *lam-2*, and *lam-3*) and ECM-related genes (*dgn-1* and *mig-6*) play important roles in embryonic morphogenesis or neuronal patterning (Huang et al. 2003; Johnson et al. 2006; Kao et al. 2006; Kawano et al. 2009; Kim and Wadsworth 2000; Vuong-Brender et al. 2017), and, therefore, their temporal suppression is important for the regulation of embryonic development. Thus, we speculate that all three corepressors serve a common role that is required for the negative regulation of genes encoding ECM- and ECM-related proteins.

## Conclusions

Using combined analyses of genetic interactions and transcriptome levels, we identified the overlapping functions among the *C. elegans* homologs of the HDAC-1 corepressors, LET-418, SIN-3, and SPR-1. Our genetic interaction and DEG analyses were consistent regarding the shared roles between *sin-3* and *spr-1* and among *sin-3, spr-1*, and *let-418*, but inconsistent regarding the *let-418-*specific role. Our terminal phenotype analyses show that *sin-3, spr-1*, and *let-418* are crucial for the progression to the middle- and late embryonic developmental stages of *C. elegans*, which is similar to previously described *hda-1(RNAi)* embryos. Finally, comparative RNA-seq analysis of these three corepressor components indicates that approximately half of upregulated and downregulated genes were associated with the SIN-3–SPR-1 group. Similarly, 10–20% of the upregulated and downregulated genes were associated with the LET-418–SIN-3–SPR-1 group. Taken together, our findings suggest that the class-I HDAC-1 corepressors, LET-418, SIN-3, and SPR-1 may cooperatively regulate the expression levels of some genes during *C. elegans* embryogenesis, or may have some similar roles but function independently within a specific cell.

## Supplementary Information

Below is the link to the electronic supplementary material.

**Supplementary Fig 1. Volcano plot of the let-418(n3536) mutant versus the WT strain, highlighting the 10 most significantly upregulated and downregulated genes related to embryogenesis. **The blue and red dots indicate downregulated and upregulated genes, respectively. A *p*-value < 0.05 was used as the threshold for statistical significance. (JPG 596 KB)

**Supplementary Fig 2. Volcano plot of the sin-3(tm1276) mutant versus the WT strain, highlighting the 10 most significantly upregulated and downregulated genes related to embryogenesis.** The blue and red dots indicate downregulated and upregulated genes, respectively. A *p-*value < 0.05 was used as the threshold for statistical significance. (JPG 787 KB)

**Supplementary Fig 3. Volcano plot of the spr-1(ok2144) mutant versus the WT strain, highlighting the 10 most significantly upregulated and downregulated genes related to embryogenesis.** The blue and red dots indicate downregulated and upregulated genes, respectively. A *p*-value < 0.05 was used as the threshold for statistical significance. (JPG 692 KB)

**Supplementary Table 1. List of the dysregulated genes observed in the let-418(n3536), sin-3(tm1276), and spr-1(ok2144) mutants, compared to levels in the WT strain.** Log_2_-normalized RNA-seq data are shown, indicating the frequencies of dysregulated genes in the *let-418(n3536)*, *sin-3(tm1276)*, and *spr-1(ok2144)* mutants divided by the corresponding expression levels in the WT strain. A *p*-value of 0.01 was used as the cut-off. (XLSX 1887 KB)

**Supplementary Table 2. List of upregulated and downregulated genes in the let-418, sin-3, and spr-1 mutants. **Expression levels (based on the RNA-seq data) are shown for upregulated genes (log_2_ fold-change > 1 and *p*-value < 0.01) and downregulated genes (log_2_ fold-change < −1 and *p*-value < 0.01). (XLSX 740 KB)

**Supplementary Table 3. GO terms that are associated with the three pathways studied. **GO terms associated with upregulated and downregulated genes that participate in embryogenic development, cell specification, cell differentiation and cellular function, gene expression, and molecular function among all three mutants (*let-418*, *sin-3*, and *spr-1*), two mutants (*sin-3* and *spr-1*), and the *let-418* mutant are shown. (XLSX 266 KB)

## Data Availability

All data and samples described in this study will be freely provided upon request.
