# Supplementary figures and images for: Overlapping and non-overlapping roles of the class-I histone deacetylase-1 corepressors LET-418, SIN-3, and SPR-1 in Caenorhabditis elegans embryonic development
Source: Genes Genomics. 2021 Mar 19;43(5):553–65. doi: 10.1007/s13258-021-01076-1 (PMC8110489; doi:10.1007/s13258-021-01076-1)

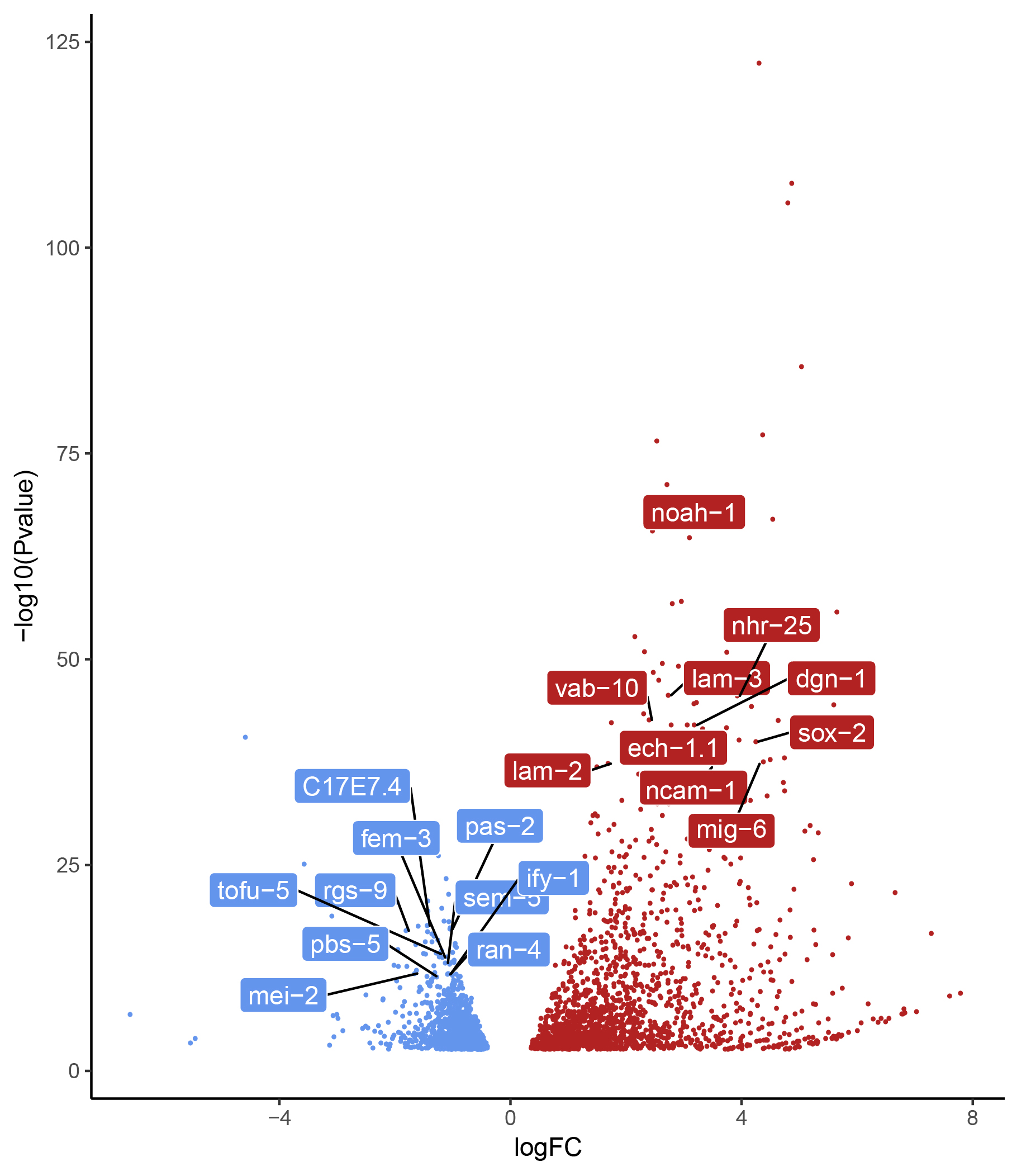

Supplement: Supplementary file 1 — Supplementary Fig 1. Volcano plot of the let-418(n3536) mutant versus the WT strain, highlighting the 10 most significantly upregulated and downregulated genes related to embryogenesis. The blue and red dots indicate downregulated and upregulated genes, respectively. A p-value < 0.05 was used as the threshold for statistical significance. (JPG 596 KB) [file 13258_2021_1076_MOESM1_ESM.jpg]

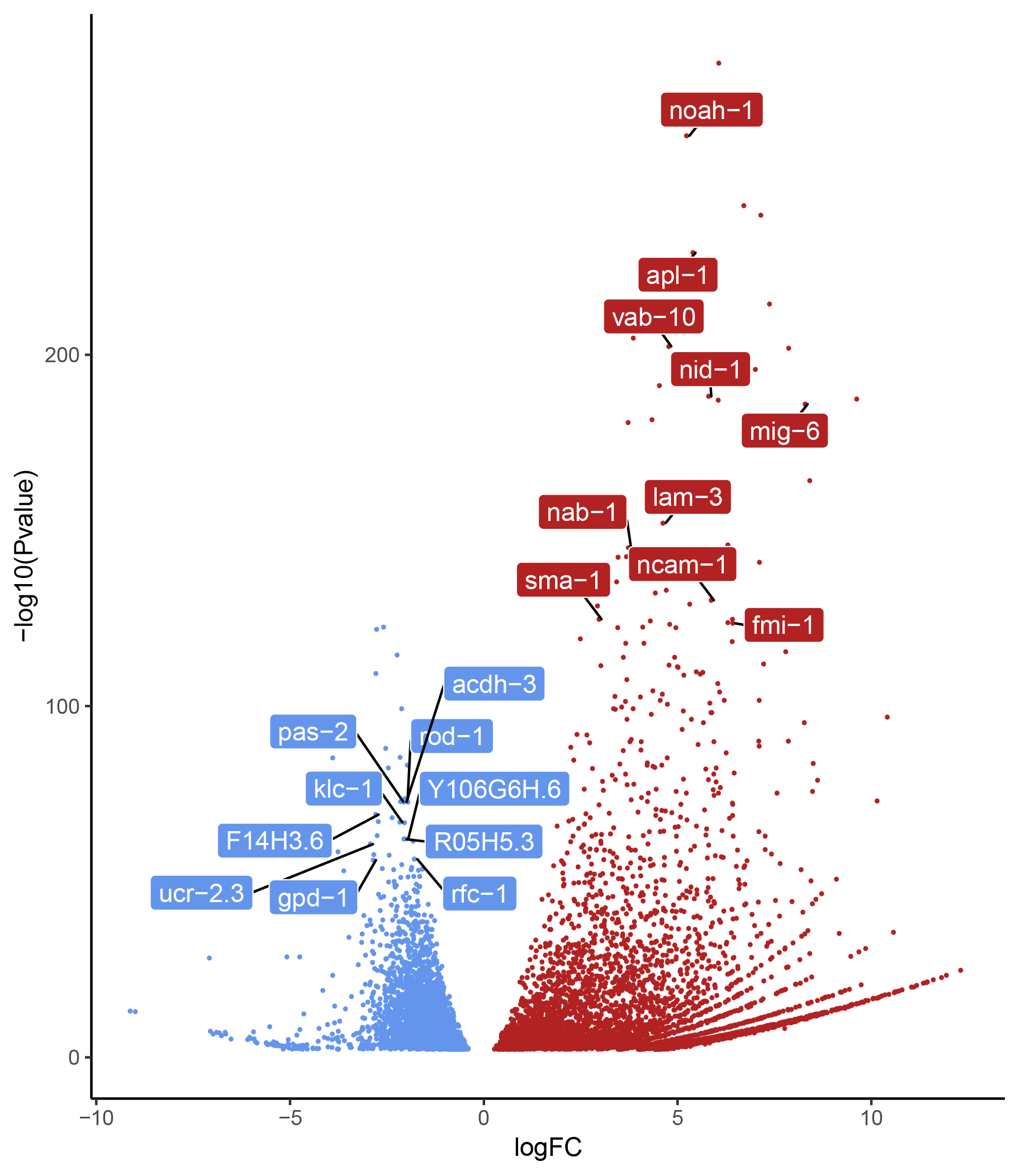

Supplement: Supplementary file 2 — Supplementary Fig 2. Volcano plot of the sin-3(tm1276) mutant versus the WT strain, highlighting the 10 most significantly upregulated and downregulated genes related to embryogenesis. The blue and red dots indicate downregulated and upregulated genes, respectively. A p-value < 0.05 was used as the threshold for statistical significance. (JPG 787 KB) [file 13258_2021_1076_MOESM2_ESM.jpg]

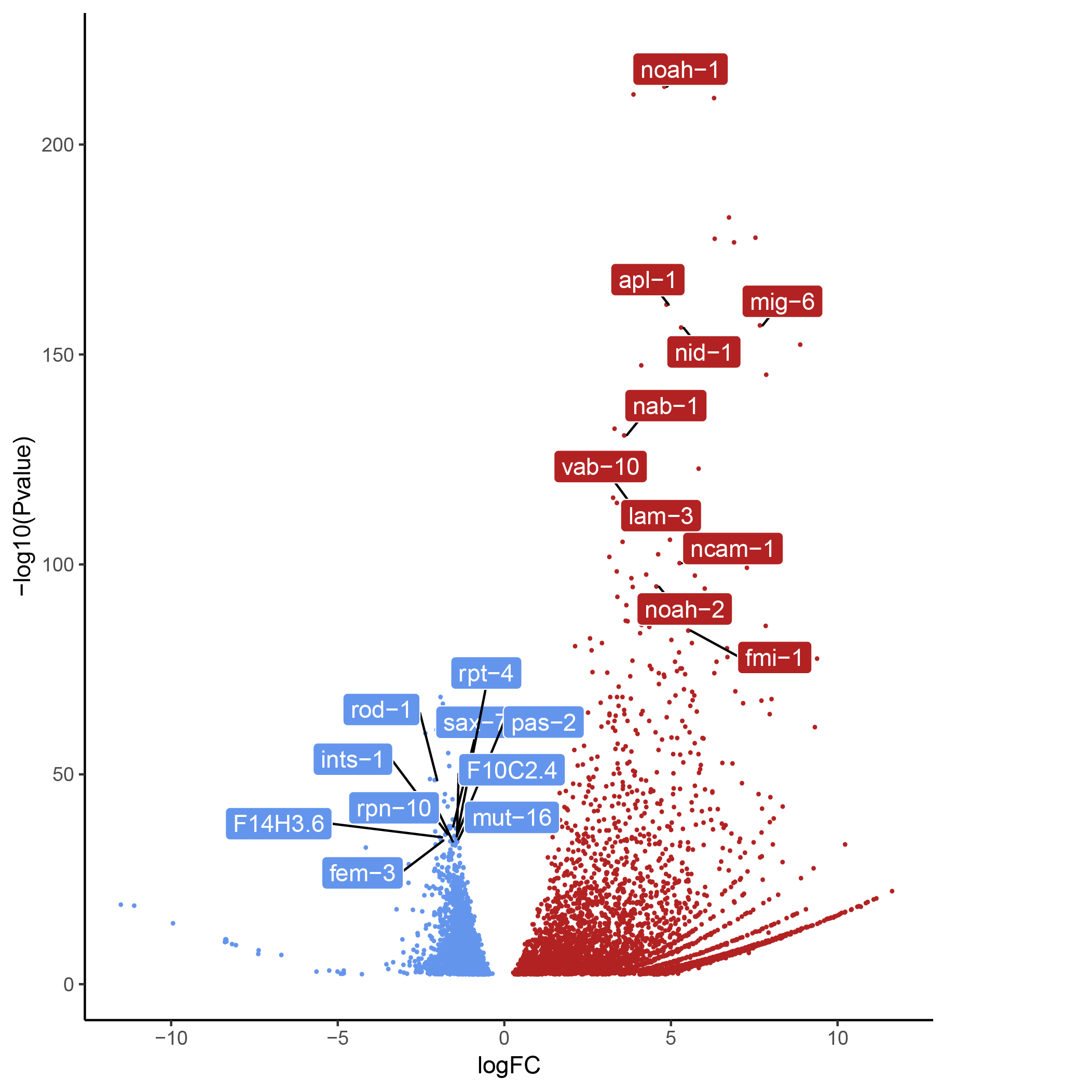

Supplement: Supplementary file 3 — Supplementary Fig 3. Volcano plot of the spr-1(ok2144) mutant versus the WT strain, highlighting the 10 most significantly upregulated and downregulated genes related to embryogenesis. The blue and red dots indicate downregulated and upregulated genes, respectively. A p-value < 0.05 was used as the threshold for statistical significance. (JPG 692 KB) [file 13258_2021_1076_MOESM3_ESM.jpg]
